# Melatonin Alleviates Chromium Toxicity in Maize by Modulation of Cell Wall Polysaccharides Biosynthesis, Glutathione Metabolism, and Antioxidant Capacity

**DOI:** 10.3390/ijms24043816

**Published:** 2023-02-14

**Authors:** Xiaoxiao Yang, Jianhong Ren, Xinyue Lin, Zhenping Yang, Xiping Deng, Qingbo Ke

**Affiliations:** 1 College of Life Sciences, Northwest A&F University, Yangling 712100, China; 2 College of Life Sciences, Shanxi Agricultural University, Taigu 030800, China; 3 College of Agronomy, Shanxi Agricultural University, Taigu 030800, China; 4 State Key Laboratory of Soil Erosion and Dryland Farming on the Loess Plateau, Institute of Soil and Water Conservation, Northwest A&F University, Yangling 712100, China; 5 Institute of Soil and Water Conservation, Chinese Academy of Sciences and Ministry of Water Resources, Yangling 712100, China

**Keywords:** melatonin, transcriptome, Cr stress, polysaccharide, glutathione

## Abstract

Melatonin, a pleiotropic regulatory molecule, is involved in the defense against heavy metal stress. Here, we used a combined transcriptomic and physiological approach to investigate the underlying mechanism of melatonin in mitigating chromium (Cr) toxicity in *Zea mays* L. Maize plants were treated with either melatonin (10, 25, 50 and 100 μM) or water and exposed to 100 μM K_2_Cr_2_O_7_ for seven days. We showed that melatonin treatment significantly decreased the Cr content in leaves. However, the Cr content in the roots was not affected by melatonin. Analyses of RNA sequencing, enzyme activities, and metabolite contents showed that melatonin affected cell wall polysaccharide biosynthesis, glutathione (GSH) metabolism, and redox homeostasis. During Cr stress, melatonin treatment increased cell wall polysaccharide contents, thereby retaining more Cr in the cell wall. Meanwhile, melatonin improved the GSH and phytochelatin contents to chelate Cr, and the chelated complexes were then transported to the vacuoles for sequestration. Furthermore, melatonin mitigated Cr-induced oxidative stress by enhancing the capacity of enzymatic and non-enzymatic antioxidants. Moreover, melatonin biosynthesis-defective mutants exhibited decreased Cr stress resistance, which was related to lower pectin, hemicellulose 1, and hemicellulose 2 than wild-type plants. These results suggest that melatonin alleviates Cr toxicity in maize by promoting Cr sequestration, re-establishing redox homeostasis, and inhibiting Cr transport from the root to the shoot.

## 1. Introduction

Chromium (Cr) is a common metal element that causes soil and water pollution [1]. In the natural environment, Cr(III) and Cr(VI) are the stable and principal forms, and Cr(VI) is more toxic than Cr(III) [2]. Cr enters farmland mainly through two pathways: natural (such as volcanoes) and anthropogenic (including paint, electroplating, tanning industries, and industrial smoke) [3,4]. Numerous studies have confirmed that higher concentrations of Cr can inhibit plant growth by reducing photosynthesis, disrupting mineral nutrient uptake, and disturbing metabolic homeostasis [5,6]. Cr(VI) can enter the human body through dietary ingestion and environmental exposure, causing various diseases such as lung cancer and gastric cancer [7]. In China, 1.26% of arable land is facing a high risk of Cr pollution. Due to the seriousness of Cr pollution, 0.13% of arable land has been abandoned [8]. Maize (*Zea mays* L.) is one of the staple crops widely used as a major food for humans and animals, and as a biofuel source [9]. More importantly, this crop is considered an accumulator of toxic metals, which is an important limiting factor for maize production and consumption [10,11]. Therefore, there is an urgent need to devise measures to alleviate Cr toxicity in maize.

To cope with heavy metal toxicity, plants have developed multiple defense strategies, such as reducing the uptake of toxic metals from the external environment, promoting the deposition of heavy metals in the apoplast, and detoxifying toxic metals inside the cell. The cell wall is the first barrier to protect against toxic metals from entering cells. Plants can enhance the heavy metal binding capacity of cell walls by regulating the biosynthesis of polysaccharides [12,13]. After heavy metals enter the cytoplasm, plants usually adopt three strategies to detoxify them: (1) chelating toxic metals with thiol group-containing compounds, such as phytochelatins (PCs) and glutathione (GSH), and then transporting the chelated complexes by ATP-binding cassette (ABC) transporters into vacuoles for compartmentalization [14,15,16]; (2) increasing the capacity of enzymatic and non-enzymatic antioxidants to mitigate metal-induced oxidative damage [17,18]; (3) activating ion transporters to pump metal out of the plasma membrane [19].

Melatonin is a pleiotropic molecule and is ubiquitous in plants and animals [20]. In 1993, melatonin was first discovered in morning glory (*Pharbitis nil*). As a multifunctional molecule, melatonin plays an essential role in regulating physiological and biochemical processes such as root development, seed germination, photosynthesis, stomatal movement, plant flowering, and post-ripening [21,22,23]. More importantly, melatonin is involved in the response to multiple abiotic stresses, including drought, salt, low/high temperatures, heavy metals, and nutrient deficits [24,25,26,27,28,29,30]. There have been some reports on the mitigating effects of melatonin on Cr toxicity. Pre-treatment with melatonin delayed Cr-induced leaf senescence by regulating chloroplast ultrastructure and stimulating osmotic adjustment in marjoram plants [31]. Seleiman et al. [32] confirmed that melatonin application significantly alleviated Cr toxicity in wheat by enhancing the antioxidant defense system and reducing Cr uptake. Additionally, seed soaking with melatonin promoted the germination of wheat seeds by improving reserve mobilization under Cr stress [33]. However, the potential mechanisms by which melatonin alleviates Cr toxicity remain poorly understood.

In recent years, transcriptome strategies have been extensively applied to elucidate heavy metal stress response mechanisms in diverse plant species, such as barley, cucumber, and rice. Genes involved in signal transduction, anthocyanin biosynthesis, GSH metabolism, redox, phenylalanine metabolism, and heavy metal transport regulated by heavy metal stress have been reported [34,35,36]. In addition, enzymes and metabolites acting downstream of these genes and involved in the regulation of biological processes in plants under stress conditions have been documented. Plants have developed multiple adaptive strategies to cope with a wide variety of stresses, including epigenetic plasticity, transcriptional network reconstruction, lipid remodeling, and physiological and metabolic reprogramming [9,37,38]. Therefore, the combined analysis of the transcriptome and physiology may improve our understanding of the regulatory mechanisms by which melatonin mitigates Cr toxicity.

Previous studies have mainly focused on the role of melatonin in protecting the photosynthetic system, reducing the Cr uptake, and increasing the antioxidant capacity [31,32,33]. In contrast, the role of melatonin in regulating the heavy metal binding ability of the cell wall has not received enough attention. Indeed, cell walls play a key role in enhancing plants’ heavy metal resistance, by preventing metals from entering the root cells [39,40]. Here, we hypothesized that melatonin could enhance the heavy metal binding ability of the cell wall by promoting the cell wall polysaccharide biosynthesis, thereby alleviating Cr toxicity in maize. Furthermore, the role of melatonin in regulating GSH metabolism and enhancing the antioxidant capacity of maize plants was also investigated. Gene expression, enzyme activities, and metabolite contents were analyzed in maize plants under Cr stress. This study provides insights into the potential mechanisms underlying the melatonin-mediated Cr stress response in maize.

## 2. Results

### 2.1. Melatonin Positively Modulates Cr Stress Resistance in Maize

**Figure 1:**
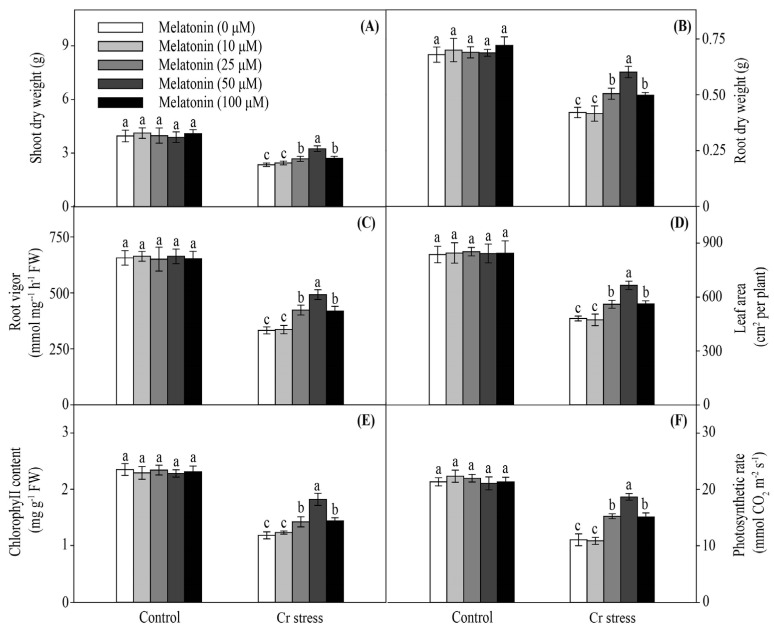
Effect of melatonin on shoot dry weight (**A**), root dry weight (**B**), root vigor (**C**), leaf area (**D**), chlorophyll content (**E**), and photosynthetic rate (**F**) in maize under Cr stress. The different letters denote significant differences at *p* < 0.05. Data are shown as mean ± SE (*n* = 3).

With no Cr treatment, no obvious difference was observed in the shoot dry weight (DW) between the melatonin-treated (MT) and non-treated (NT) plants. Cr stress significantly decreased the shoot DW. In contrast, the shoot DW was increased by melatonin treatment, which reached the highest level at 50 μM/L melatonin (Figure 1A). Similarly, MT plants showed higher root DW than NT plants (Figure 1B). Consistently, the root vigor, leaf area, chlorophyll content, and photosynthetic rate of MT plants were higher than those of NT plants (Figure 1C–F). Taken together, these results indicate that melatonin can alleviate Cr toxicity in maize.

### 2.2. Melatonin Cannot Influence the Cr Uptake in Maize

**Figure 2:**
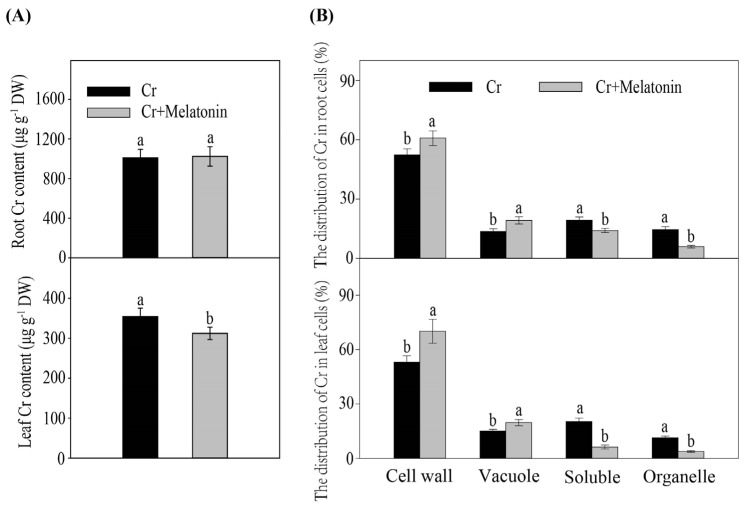
Effect of melatonin on Cr content (**A**) and its subcellular distribution (**B**) in maize roots and leaves during Cr stress. Different letters in one measure (cell wall, vacuole, soluble, or organelle) denote significant differences at *p* < 0.05. Data are shown as mean ± SE (*n* = 3).

Cr stress considerably increased the Cr content in both leaves and roots. Melatonin application markedly decreased the Cr content in leaves. However, in the MT plants, Cr contents in the roots were similar to those of the NT plants (Figure 2A). Furthermore, we assessed the subcellular distribution of Cr in the roots and leaves of maize. The Cr levels in cell walls and vacuoles were markedly higher in MT plants than in NT plants during Cr stress, while the Cr levels in the cytoplasm and organelle were lower in MT plants than in NT plants (Figure 2B).

### 2.3. Melatonin and Cr Treatments Induce Transcriptome Reprogramming in Maize

**Figure 3:**
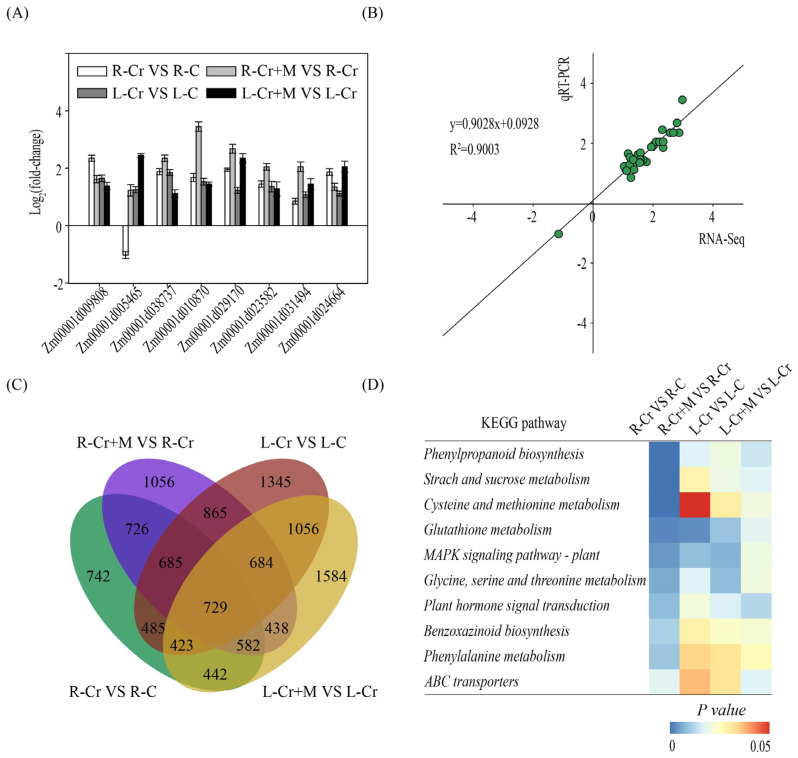
Melatonin and Cr induced changes in gene transcripts in maize roots and leaves. (**A**) Transcriptional levels of genes (selected from transcriptome) were analyzed by the qRT-PCR. Data are shown as mean ± SE (*n* = 3). R-C, control roots; L-C, control leaves; R-Cr, Cr-treated roots; L-Cr, Cr-treated leaves; R-Cr+M, Cr+melatonin-treated roots; L-Cr+M, Cr+melatonin-treated leaves. (**B**) Correlation analysis between transcriptome (*x*-axis) data and qRT-PCR (*y*-axis) results. (**C**) Venn diagram representing the overlap of DEGs in different comparisons. (**D**) KEGG pathway enrichment analysis for DEGs. The color scale represents the significance level.

To further clarify the possible mechanism of the alleviating effect of melatonin on Cr stress, transcriptome analysis was performed on the leaves and roots of maize in the Cr and Cr+Melatonin (50 μM) treatments. A high correlation was observed between RNA-Sequencing and qRT-PCR data, confirming the accuracy of the transcriptome results (Figure 3A,B). In the Cr vs. Control and Cr+Melatonin (50 μM) vs. Cr comparisons, 4814 and 5765 differentially expressed genes (DEGs; |log_2_fold change| > 1) were identified in roots, and 6272 and 5938 DEGs were identified in leaves, respectively (Figure 3C). Subsequently, we performed a Kyoto Encyclopedia of Genes and Genomes (KEGG) pathway enrichment analysis to identify the metabolic pathways potentially affected by melatonin during Cr stress (Figure 3D). These pathways were mainly related to starch and sucrose metabolism, cysteine and methionine metabolism, MAPK signaling, plant hormone signal transduction, glutathione metabolism, and ABC transports.

### 2.4. Melatonin Enhances Cr Accumulation in the Cell Walls in Maize

**Figure 4:**
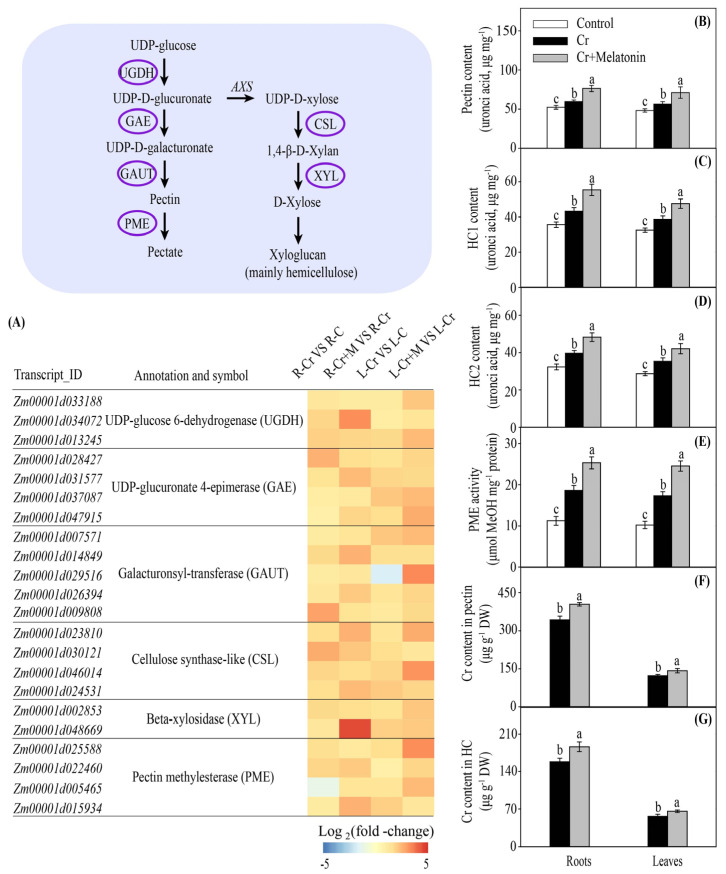
Melatonin and Cr induced changes in gene transcripts involved in cell wall polysaccharide metabolism in maize roots and leaves (**A**). The color scale represents log_2_ (fold-change). The effect of melatonin on the uronic acid contents of cell wall polysaccharides ((**B**), pectin; (**C**), hemicellulose 1; (**D**), hemicellulose 2), pectin methylesterase (PME) activity (**E**), Cr contents in the cell wall pectin (**F**), and the Cr contents in the cell wall hemicellulose (**G**) in maize roots and leaves under Cr stress. Different letters in one measure (roots or leaves) denote significant differences at *p* < 0.05. Data are shown as mean ± SE (*n* = 3).

Plant cell walls are mainly composed of cellulose, hemicelluloses (HCs), pectins, and some proteins. Among these components, HCs and pectins are considered to be the two major components for binding heavy metals [41]. During Cr stress, genes involved in pectin and hemicellulose biosynthesis were significantly upregulated in the roots and leaves, including *UDP-glucose 6-dehydrogenase* (*UGDH*), *UDP-glucuronate 4-epimerase* (*GAE*), *galacturonsyl-transferase* (*GAUT*), *cellulose synthase-like* (*CSL*), and *beta-xylosidase* (*XYL*). Melatonin application further enhanced the transcription of these genes (Figure 4A). Consistently, melatonin treatment significantly enhanced the contents of pectin, hemicellulose 1 (HC1), and hemicellulose 2 (HC2) in leaves and roots (Figure 4B–D).

The degree of pectin methylation, which is regulated by pectin methylesterase (PME), can also influence the binding capacity of pectin [42]. Under Cr stress, genes encoding PME were significantly upregulated, and this increase was further augmented by melatonin application (Figure 4A). Consistently, the exogenous addition of melatonin enhanced the PME activities in roots and leaves (Figure 4E). We next examined the Cr content in cell wall pectin and HCs. The Cr concentrations in the cell wall pectin in the roots and leaves of MT plants were 17.7% and 16.5% higher than those of NT plants, respectively (Figure 4F,G). Collectively, these data suggest that melatonin enhances the capacity for binding Cr to the cell wall.

### 2.5. Melatonin Increases the Glutathione and Phytochelatin Contents in Maize

**Figure 5:**
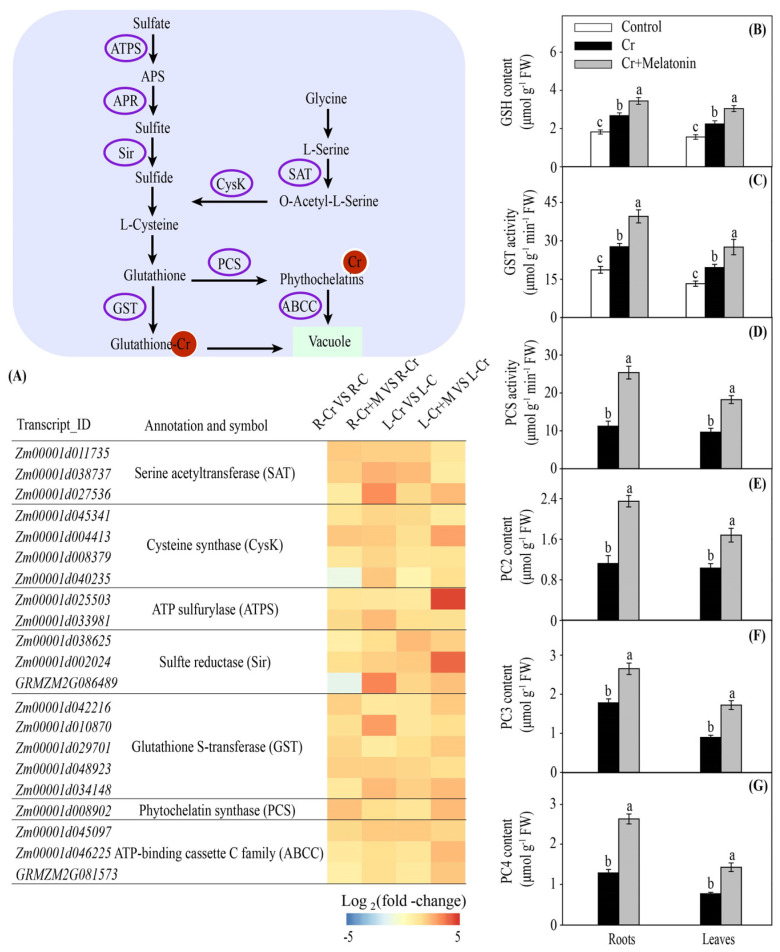
Melatonin and Cr induced changes in gene transcripts involved in glutathione (GSH) metabolism in maize roots and leaves (**A**). The color scale represents log_2_ (fold-change). The effect of melatonin on glutathione (GSH) content (**B**), glutathione S-transferase (GST) activity (**C**), phytochelatin synthase (PCS) activity (**D**), and phytochelatins (PCs) contents (**E**–**G**) in maize roots and leaves under Cr stress. Different letters in one measure (roots or leaves) denote significant differences at *p* < 0.05. Data are shown as mean ± SE (*n* = 3).

GSH is known to possess a strong capacity for toxic metal detoxification owing to the high affinity of its sulfhydryl group. Cysteine metabolism is essential for GSH synthesis in plants [43]. Under Cr stress, melatonin markedly increased the transcription levels of genes involved in cysteine synthesis in both leaves and roots, including *serine acetyltransferase* (*SAT*), *cysteine synthase* (*CysK*), *ATP sulfurylase* (*ATPS*), *adenosine 5′-phosphosulfate reductase* (*APR*), and *sulfate reductase* (*Sir*) (Figure 5A). The GSH content increased by 28.7% and 35.6% in the roots and leaves of MT plants, respectively, compared to that in the NT plants (Figure 5B). Moreover, we found that *Glutathione S-transferase* (*GST*) genes were upregulated by melatonin (Figure 5A). Consistently, melatonin application significantly increased the GST activity in roots and leaves during Cr stress (Figure 5C).

PC is a low-molecular-weight thiol that, similarly to its precursor GSH, exerts a vital role in the detoxification of heavy metals. Under Cr stress, the phytochelatin synthase (*PCS*) gene was significantly upregulated in the roots and leaves, and this tendency was further increased by melatonin application (Figure 5A). Consistently, the PCS activities in the roots and leaves were significantly enhanced by melatonin treatment (Figure 5D). We further assessed the concentration of PCs in maize plants. The concentrations of phytochelatin 2 (PC2), phytochelatin 3 (PC3), and phytochelatin 4 (PC4) in MT plants were 109.8%, 49.0%, and 105.9% higher in the roots and 63.1%, 92.3%, and 84.4% higher in the leaves, respectively, compared to those of NT plants (Figure 5E–G). Moreover, melatonin application significantly enhanced the expression of *ZmABCC* in the roots and leaves (Figure 5A). Collectively, these findings indicate that melatonin promotes metal-chelating compound biosynthesis under Cr stress.

### 2.6. Melatonin Increases Antioxidant Capacity in Maize

**Figure 6:**
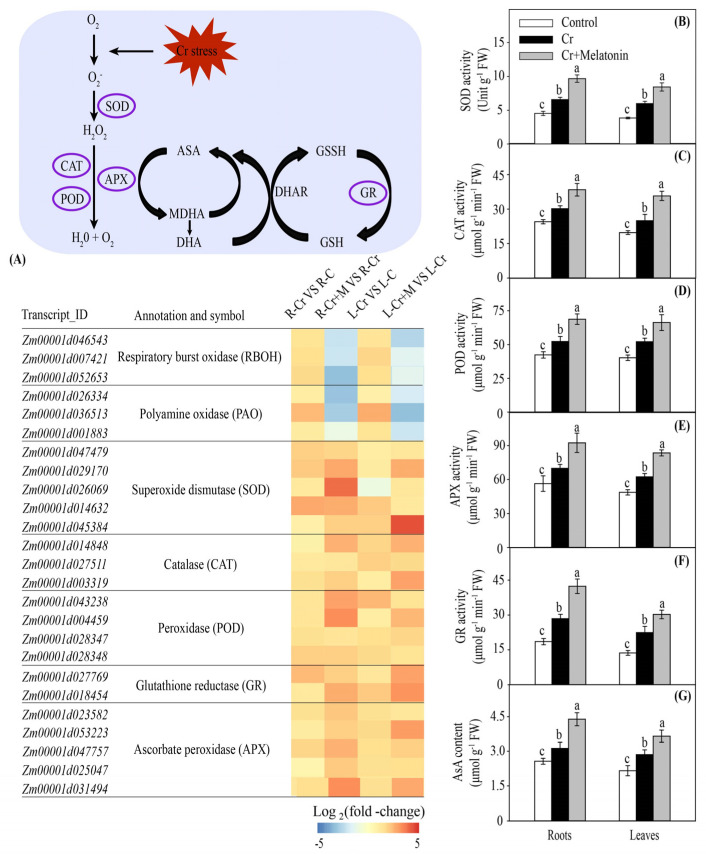
Melatonin and Cr induced transcript changes of genes in antioxidant enzyme biosynthesis in maize roots and leaves (**A**). The color scale represents log_2_ (fold-change). The effect of melatonin on superoxide dismutase (SOD) activity (**B**), catalase (CAT) activity (**C**), peroxidase (POD) activity (**D**), ascorbate peroxidase (APX) activity (**E**), glutathione reductase (GR) activity (**F**), and ascorbate (AsA) content (**G**) in maize roots and leaves during Cr stress. Different letters in one measure (roots or leaves) denote significant differences at *p* < 0.05. Data are shown as mean ± SE (*n* = 3).

**Figure 7:**
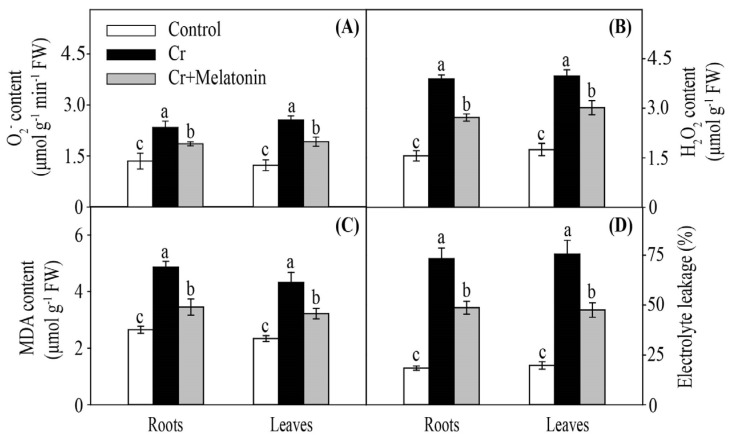
Effect of melatonin on O_2_^•−^ content (**A**), hydrogen peroxide (H_2_O_2_) content (**B**), Malondialdehyde (MDA) content (**C**), and electric leakage (**D**) in maize roots and leaves during Cr stress. Different letters in one measure (roots or leaves) denote significant differences at *p* < 0.05. Data are shown as mean ± SE (*n* = 3).

The genes involved in reactive oxygen species (ROS) production were significantly upregulated in the roots and leaves under Cr stress, including *respiratory burst oxidase* (*RBOH*) and *polyamine oxidase* (*PAO*). Importantly, melatonin application reversed this harmful effect (Figure 6A). Consistently, melatonin treatment markedly reduced the levels of superoxide anion radical (O_2_^•−^) and hydrogen peroxide (H_2_O_2_) in the roots and leaves during Cr stress (Figure 7A,B). Similarly, the malondialdehyde (MDA) content and electric leakage (EL) decreased by 29.0% and 33.6% in the roots and 25.7% and 37.2% in the leaves, respectively, in MT plants compared to those of NT plants (Figure 7C,D).

Melatonin markedly enhanced the transcription of genes involved in antioxidant enzyme synthesis, including *superoxide dismutase* (*SOD*), *catalase* (*CAT*), *peroxidase* (*POD*), *ascorbate peroxidase* (*APX*), and *glutathione reductase* (*GR*) (Figure 6A). Consistently, the exogenous addition of melatonin substantially enhanced the activities of antioxidant enzymes in the roots and leaves under Cr stress (Figure 6B–F). Moreover, melatonin application markedly increased the ascorbate (AsA) content in the roots and leaves (Figure 6G). These findings indicate that MT plants display a higher antioxidant capacity than NT plants under Cr stress.

### 2.7. Melatonin Increases Endogenous Melatonin Content in Maize

**Figure 8:**
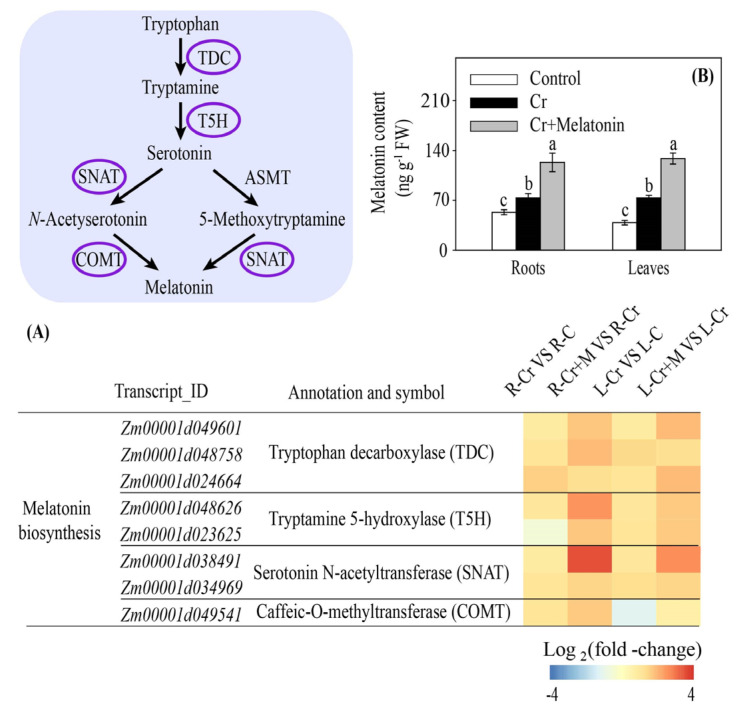
Melatonin and Cr induced changes in gene transcripts involved in melatonin biosynthesis in maize roots and leaves (**A**). The effect of melatonin on endogenous melatonin content in maize under Cr stress (**B**). Different letters in one measure (roots or leaves) denote significant differences at *p* < 0.05. Data are shown as mean ± SE (*n* = 3).

Cr stress substantially enhanced the transcription of genes, including tryptophan decarboxylase (TDC), tryptamine 5-hydroxylase (T5H), serotonin N-acetyltransferase (SNAT), and caffeic-O-methyltransferase (COMT), involved in melatonin biosynthesis in both the roots and leaves. Melatonin application further increased this effect (Figure 8A). Consistently, melatonin treatment significantly enhanced the endogenous melatonin content in leaves and roots (Figure 8B).

### 2.8. Modulation of Melatonin Content in Arabidopsis Confers Enhanced Cr Stress Tolerance

**Figure 9:**
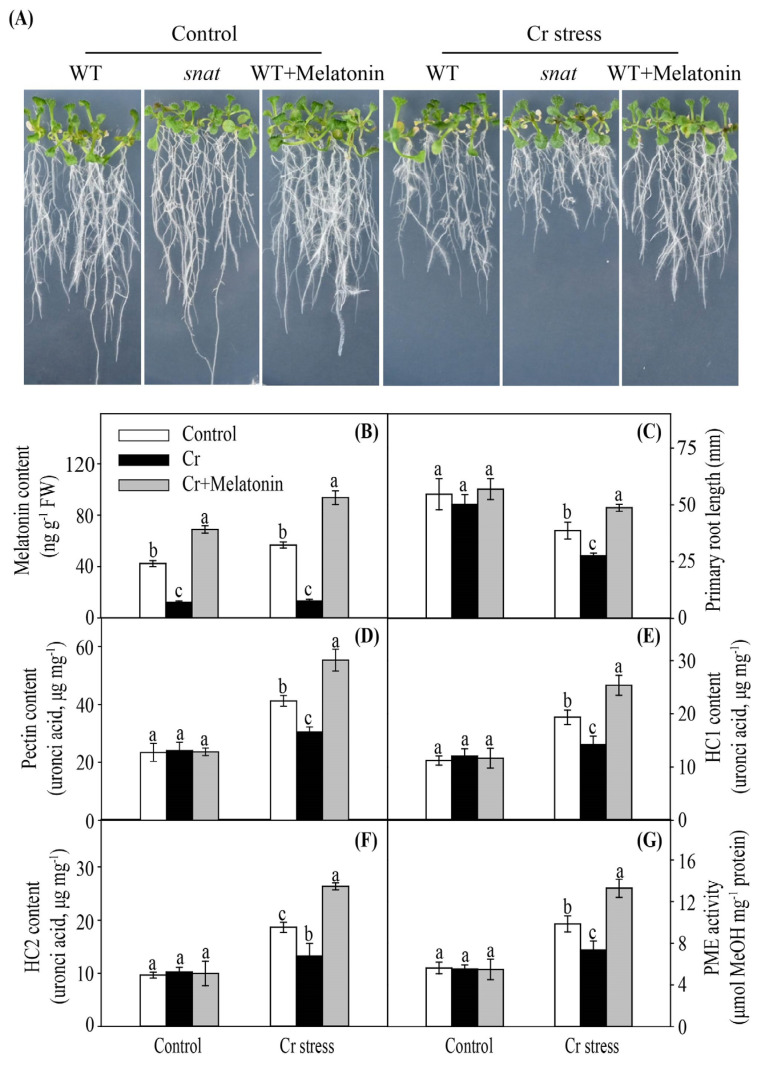
Phenotypes of *Arabidopsis* after Cr stress treatment (**A**). The modulation of melatonin affects endogenous melatonin content (**B**), root growth (**C**), uronic acid contents of cell wall polysaccharides ((**D**), Pectin; (**E**), hemicellulose 1; (**F**), hemicellulose 2), and pectin methylesterase (PME) activity (**G**) in *Arabidopsis* roots under Cr stress. Different letters in one measure (control or Cr stress) denote significant differences at *p* < 0.05. Data are shown as mean ± SE (*n* = 3).

To further confirm the positive role of melatonin in regulating the heavy metal binding ability of the cell wall, a melatonin biosynthesis-defective *snat* mutant was employed. Under Cr stress, exogenous melatonin-treated wild-type (WT) plants grew better and displayed a higher level of endogenous melatonin, while the *snat* mutants grew worse and showed a lower level of endogenous melatonin in comparison to non-treated WT plants (Figure 9A,B). Melatonin-treated WT plants displayed higher root length, while the *snat* mutants showed lower root lengths than non-treated WT plants (Figure 9C). Moreover, melatonin-treated WT plants displayed higher levels of pectin, HC1, HC2, and PME, while the *snat* mutants showed lower levels of pectin, HC1, HC2, and PME in comparison to non-treated WT plants (Figure 9D–G).

## 3. Discussion

Heavy metal stress seriously inhibits plant growth and development [5,44]. Previously, melatonin was shown to promote the growth of diverse plant species, including watermelon, tomato, cucumber, and wheat, under heavy metal stress [45,46,47,48]. In agreement with previous studies, our results showed that Cr stress significantly inhibited plant growth (Figure 1A). In contrast, the exogenous application of melatonin under Cr stress partly alleviated Cr-induced growth inhibition and significantly decreased the Cr content in leaves. Previous studies have shown that melatonin can decrease the metal content in the leaves of wheat [46] and tomatoes [49]. In plants, heavy metal ATPase2 (HMA2) and heavy metal ATPase4 (HMA4) are suggested to be involved in the loading of heavy metals into the xylem and their subsequent translocation from the root to the shoot [50]. In this study, MT plants exhibited reduced expression levels of ZmHMA2 and ZmHMA4, indicating reduced Cr translocation (Supplemental Table S1). The role of melatonin in the inhibition of HMA2 expression has been emphasized in other plant species, such as tobacco, under Cd stress [51]. All these findings suggest that melatonin could inhibit Cr transport from the root to the shoot, preventing Cr from reaching photosynthetic leaf tissues. However, the Cr content in the roots was not affected by the melatonin treatment (Figure 2A), indicating that the alleviative effect of melatonin was not due to a reduction in Cr uptake from the external environment. The transcriptomic and physiological results obtained in our research deepen our knowledge about the regulatory mechanism by which melatonin mitigates Cr toxicity.

### 3.1. Melatonin Enhances Cr Binding Capacity of Cell Walls in Maize

As the first barrier in contact with heavy metals, the cell walls of the roots are the predominant sites for binding the heavy metals and act as a barrier to prevent the entry of external heavy metals into the cytoplasm [52]. For instance, assays of the subcellular distribution of cadmium in *Arabidopsis thaliana* [53], aluminum in alfalfa [54], and lead in *Medicago truncatula* [55] suggest that the cell wall is the main sink for toxic metals. Cell wall polysaccharides, especially pectin and hemicelluloses (HCs), are major sites for metal ion binding. A higher content of pectins and/or HCs results in greater toxic metal accumulation in the cell walls of rapeseed and rice [40,56]. Therefore, factors that could affect cell wall components may influence the metal binding capacity of the cell walls. In this study, we found that melatonin-producing and MT plants displayed higher Cr concentrations in cell wall pectin and HCs than NT plants (Figure 4F,G). These findings suggest that increased melatonin production enhances the Cr binding capacity of cell walls by regulating pectin and HCs biosynthesis.

The degree of pectin methylation is also an important factor in determining the heavy metal binding capacity of the cell wall. The degree of pectin methylation is widely recognized to be negatively correlated with the accumulation of metals in the cell wall (such as aluminum and cadmium) [39,42]. The demethylation of pectin is regulated by PME. Wu et al. [57] found that the cadmium-tolerant oilseed rape cultivar showed higher PME activity in the leaves. In the present study, MT plants showed higher PME activities in the roots than NT plants (Figure 4E), suggesting that MT plants could provide more negatively charged carboxyl groups to bind to Cr. Previous studies have shown that melatonin can enhance *PME* expression in copper-treated plants [34]. These findings suggest that melatonin could increase the negatively charged sites in the cell wall by enhancing pectin content and improving PME activity, and then advance Cr accumulation in the cell wall.

### 3.2. Melatonin Promotes Cr Chelation in Maize

GSH is a member of the ascorbate–glutathione cycle and serves as a metal chelator [15,43]. It has been confirmed that enhanced synthesis of GSH alleviates cadmium toxicity in rice [58]. Additionally, an exogenous supply of GSH can alleviate zinc toxicity in safflower by enhancing PC synthesis, activating antioxidant enzymes, and regulating the AsA-GSH cycle [59]. In this study, melatonin addition substantially increased the GSH content in roots and leaves during Cr stress (Figure 5B). Furthermore, we found that the gene transcriptional levels and enzymatic activity of GST were enhanced by melatonin when exposed to Cr stress (Figure 5A,C). In plants, the GST plays an essential role in cellular homeostasis and glutathione metabolism by catalyzing the binding of heavy metals to reduced GSH and decreasing metals inside the cytosol [60,61]. Siddiqui et al. (2020) [62] demonstrated that melatonin treatment increases the GST activity in arsenic-treated *Vicia faba* plants.

PC is a derivative of GSH, which participates in the sequestration of heavy metals [14,63]. Overexpression of genes involved in PCS synthesis has been demonstrated to increase heavy metal stress tolerance in Indian mustard and tobacco [64,65]. Numerous studies have shown that melatonin can increase the PC content in tomatoes [66] and rice [67] under heavy metal stress. In the present research, the gene transcription level and enzyme activity of PCS were enhanced by melatonin in maize plants under Cr stress (Figure 5A,D). In addition, the concentrations of PC2, PC3, and PC4 were significantly increased by melatonin treatment (Figure 5E–G).

In plants, heavy metal-PC complexes are transported into vacuoles by ABC transporters (mainly the ABCC subfamily) for compartmentalization [16,68]. It has been demonstrated that ABCC transporters are located on the vacuole membrane and are involved in metal-PC complex transport, such as AtABCC1 and AtABCC2 in *Arabidopsis* [69] and OsABCC1 in rice [70]. In this study, the expression levels of ZmABCC genes in the roots and leaves were significantly enhanced by melatonin application (Figure 5A). Overall, it is likely that melatonin could increase the content of thiol-containing compounds (e.g., PCs and GSH) and *ABCC* gene expression, thus reducing the heavy metals inside the cytoplasm.

### 3.3. Melatonin Increases Antioxidant Capacity in Maize

During normal growth conditions, there is a dynamic balance between ROS production and scavenging. However, heavy metal stress can break the equilibrium relationship between the production and removal of ROS, resulting in excessive ROS accumulation [17,71]. It has been confirmed that melatonin can mitigate heavy metal stress-induced oxidative stress in plants, such as cadmium, nickel, and arsenic [48,72,73]. In agreement with previous studies, our results demonstrated that the levels of O_2_^•−^ and H_2_O_2_ in the roots and leaves were significantly decreased by melatonin treatment under Cr stress (Figure 7A,B). The MDA and EL are key indicators of the degree of lipid peroxidation and integrity of cell membranes, respectively [74]. Overaccumulation of ROS in cells could cause lipid peroxidation and further destroy the integrity of the cell membrane [75]. In this study, melatonin markedly reduced the contents of MDA and EL in both leaves and roots under Cr stress (Figure 7C,D), indicating that melatonin plays a vital role in reducing lipid peroxidation and maintaining cell membrane integrity.

To prevent damage from ROS, plants have evolved antioxidant (enzymatic and non-enzymatic) defense systems [76,77]. Numerous previous studies have established that melatonin can enhance the activities of antioxidant enzymes when exposed to toxic metal stress, including SOD, CAT, and POD [51,78]. Consistently, melatonin markedly increased the activity of antioxidant enzymes in maize plants (Figure 6B–F). Moreover, Okant and Kaya [79] confirmed that NO was involved in melatonin-mediated lead stress tolerance by activating antioxidant enzymes in maize plants. Additionally, the AsA–GSH cycle exerts a vital role in the mitigation of metal-induced oxidative damage [80]. In this study, the contents of GSH and AsA in the roots and leaves were substantially increased by melatonin application (Figure 5B and Figure 6G). These results indicated that melatonin could alleviate Cr-induced oxidative stress by enhancing the capacity of enzymatic and non-enzymatic antioxidants.

## 4. Materials and Methods

### 4.1. Experimental Design

Maize seeds (*Zea mays* L. “QS101”, a Cr-sensitive cultivar) were sterilized in 1% sodium hypochlorite and then washed three times using sterilized H_2_O. Seeds were germinated on wet filter papers in the dark for 3 days at 25 °C. Subsequently, maize plants were transplanted into a plastic container filled with 6 L of Hoagland solution. Three weeks later, foliar portions of maize plants were sprayed with 0, 10, 25, 50, or 100 μM melatonin. The melatonin solutions were prepared by dissolving melatonin in ethanol. In each pot, 50 mL of melatonin solution or water was sprayed directly into foliar portions in a single application. After melatonin treatment for 12 h, 100 μM of K_2_Cr_2_O_7_ (analytical grade) was added to the nutrient solution to induce Cr stress. This study included ten treatments: (1) 0 μM melatonin (Control), (2) 10 μM melatonin, (3) 25 μM melatonin, (4) 50 μM melatonin, (5) 100 μM melatonin, (6) 100 μM K_2_Cr_2_O_7_, (7) 100 μM K_2_Cr_2_O_7_ + 10 μM melatonin, (8) 100 μM K_2_Cr_2_O_7_ + 25 μM melatonin, (9) 100 μM K_2_Cr_2_O_7_ + 50 μM melatonin, and (10) 100 μM K_2_Cr_2_O_7_ + 100 μM melatonin. Each treatment contained 5 plastic boxes and each box contained 12 seedlings. The hydroponic solution was changed once every two days. After seven days of Cr stress treatment, the youngest, fully expanded leaves and roots were collected. Roots or leaves were sampled from five plants located at five different boxes and pooled together to comprise an independent replicate. Each treatment consisted of three independent biological replicates. The seedlings were grown in a growth chamber at 26 °C under the photoperiod of 12 h light/12 h dark.

*Arabidopsis thaliana*, Columbia ecotype, served as the control. The *snat* mutant has been described previously [81,82]. Seeds were sterilized in 8% sodium hypochlorite and then washed five times using sterilized H_2_O. Seeds were incubated for 3 days at 4 °C and planted on Murashige and Skoog (MS) solid medium (0.7% agar, 3% sucrose) in the growth chamber. Seven days later, seedlings were treated with 0 or 50 μM melatonin for 12 h. After melatonin treatment, 100 μM of K_2_Cr_2_O_7_ was added to the MS solid medium to induce Cr stress. After 24 h, all plants were transplanted into a fresh MS solid medium for another 3 days. The growth chamber was set at 120 µmol m^−2^s^−1^ photosynthetic photon flux density, 23 °C temperature, and 65% humidity under the photoperiod of 16 h light/8 h dark. The experiment scheme is presented in Supplemental Figure S1.

### 4.2. Determination of Dry Weight, Root Vigor, Leaf Area, Chlorophyll Content, and Photosynthetic Rate

Maize roots and shoots were collected separately, oven-dried for 72 h at 70 °C, and weighed to determine the dry weight (DW). Root vigor was assessed according to Zhang et al. [83]. Leaf area was determined using a leaf area meter (CI-203, CID Inc., Camas, WA, USA). The chlorophyll content was determined as reported in [84]. Fresh leaves (0.2 g) were extracted using 90% acetone in the dark for 24 h. The homogenates were centrifuged for 10 min at 6000 rpm and the absorbance of the supernatant was measured at 645 and 663 nm with a spectrophotometer (UV-2600, Shimadzu, Kyoto, Japan). The photosynthetic parameters of the youngest, fully expanded leaves were measured between 10:00 and 11:00 AM by a portable photosynthesis system (LI-6400, LI-COR, Lincoln, NE, USA). During measurements, the leaf chamber was set at 1000 µmol m^−2^s^−1^ photosynthetic photon flux density, 25 °C leaf temperature, 500 µmol s^−1^ flow rate, and 50% humidity. Three replicates were analyzed per treatment.

### 4.3. Determination of Cr Content

Fresh samples (5 g) of leaves and roots were separated into four fractions: soluble, organelle, vacuole, and cell wall, as reported previously [49]. Briefly, samples were homogenized using a buffer containing 1 mM dithiothreitol (DTT), 250 mM sucrose, 1.0% *w*/*v* polyvinylpolypyrrolidone (PVPP), 50 mM Tris-HCl, and 5 mM ascorbic acid. The extracts were filtered through an 80 μm nylon cloth and then centrifuged to obtain different fractions. Separated cell fractions, leaves, and roots (0.2 g) were digested at 180 °C using a 5:1 (*v*/*v*) mixture of HNO_3_ and HClO_4_. The Cr contents in all samples were assayed using an inductively coupled plasma emission spectrometer (Agilent 725, Agilent Technologies, Santa Clara, CA, USA). Three replicates were contained for each treatment.

### 4.4. Transcriptome Sequencing and qRT-PCR Analyses

Seven days after Cr treatment, the roots and youngest fully expanded leaves of maize in the control, Cr, and Cr+Melatonin (50 μM) treatments were collected for RNA sequencing. Total RNA was extracted from the samples with a Direct-zol RNA MiniPrep kit (Zymo Research, Irvine, CA, USA) according to the manufacturer’s protocol. The integrity (RIN) and purity of the RNA samples were evaluated with a Bioanalyzer RNA 6000 Nano kit (Agilent Technologies, Santa Clara, CA, USA) and Qubit RNA Assay Kit (Life Technologies, New York, NY, USA), respectively, and the RNA concentration was measured using a Nanodrop 2000c spectrophotometer (Thermo Scientific, Wilmington, MA, USA). RNA samples with OD260/230 > 2.0, OD260/280 > 1.8, and RIN > 8.0 were retained for further analysis. Ribosomal RNA was removed using the Ribo-Zero Gold depletion Kit (Illumina, San Diego, USA), and the ethanol precipitation method was used to purify the rRNA-free residue. For RNA sequencing library generation, the TruSeq RNA Sample Preparation Kit V2 (Illumina, San Diego, CA, USA) was used. The sequencing library quality was evaluated using a 2100 Bioanalyzer (Agilent Technologies, Santa Clara, CA, USA), and sequencing was conducted on an Illumina HiSeq 2500 system (Majorbio, Shanghai, China). After the removal of low-quality reads, the clean reads were mapped to the reference genome of maize (B73). The Cufflinks software was used to identify the differentially expressed genes (DEGs, false discovery rate < 0.05 and log_2_ fold change > 1), and the KOBAS software was used to identify the KEGG pathways enriched among the DEGs [85]. All DEGs are listed in Table S2. Four comparisons were performed, including R-Cr/R-C, R-Cr+M/R-Cr, L-Cr/L-C, and L-Cr+M/L-Cr. R-C, Control roots; L-C, Control leaves; R-Cr, Cr-treated roots; L-Cr, Cr-treated leaves; R-Cr+M, Cr+melatonin-treated roots; L-Cr+M, Cr+melatonin-treated leaves. Raw sequencing reads were stored in the National Center for Biotechnology Information (PRJNA913565). Eight genes were selected to verify the transcriptome results using qRT-PCR, as described previously. The primers used are listed in Supplemental Table S3, and ZmUbi-2 was used as the reference gene. The relative expression levels of selected genes were calculated by the 2^−ΔΔCt^ method.

### 4.5. Metabolite Content Assays

The uronic acid content of the cell wall polysaccharides was assessed, as reported by Yuan et al. [32]. Fresh samples (0.5 g) were homogenized with a 75% ethanol solution. The suspensions were centrifuged for 10 min at 5000× *g*. Subsequently, the obtained pellet was successively rinsed with acetone, methanol, trichloromethane (1:1, *v*/*v*), and methanol. Cell wall materials in the pellet were separated into different fractions. Fractions of HC1, HC2, and pectin were collected using KOH (4%), KOH (24%), and ammonium oxalate buffer (0.5%), respectively.

The GSH and PCs were analyzed using HPLC (Agilent 1260, Agilent Technologies, Santa Clara, USA), as described previously [86]. Fresh tissues (1 g) of leaves and roots were extracted using a buffer containing 6.3 mM diethylene triamine-pentaacetic acid (DTPA) and 5% (*w*/*v*) 5-sulfosalicylic acid. The homogenates were centrifuged for 10 min at 15 °C at 10,000× *g*. Samples were then filtered through a 0.45 µm filter. The GSH and PCs were separated on a reverse-phase Purospher C18 column (Merck) using an acetonitrile gradient (0–26%) containing 0.05% trifluoroacetic acid (TFA) at a flow rate of 0.7 mL/min. Sulfhydryl compounds were detected at 412 nm after post-column derivatization using Ellman’s reagent. All thiol standards were purchased from Sigma-Aldrich, St. Louis, MO, USA.

The O_2_^•−^ content was analyzed, as reported previously [48]. Frozen tissues of roots and leaves (0.5 g) were homogenized in a phosphate buffer (65 mM), and the samples were then centrifuged for 10 min at 5000× *g*. Subsequently, the supernatant was mixed with potassium phosphate buffer (50 mM) and hydroxylamine hydrochloride (10 mM). The incubated solution was mixed with 7 mM naphthylamine and 17 mM sulfanilamide. After incubation for 30 min at 25 °C, the absorbance of the mixture was monitored at 530 nm. The O_2_^•−^ content was calculated using the standard curve of NaNO_2_. The H_2_O_2_ content was assayed as described in [87]. The MDA content was determined as reported by Okant and Kaya [79]. Briefly, fresh samples (0.2 g) were homogenized with 0.1% (*w*/*v*) trichloroacetic acid (TCA). The suspensions were centrifuged for 5 min at 12,000× *g* at 4 °C. The absorbance of the supernatant was measured at 532 nm with a spectrophotometer (UV-2600). The EL was analyzed using a conductivity instrument (LC116, Mettler Toledo Instruments, Shanghai, China), as described by Abo Gamar et al. [74]. The assays for the AsA content were conducted as described in [80]. Frozen tissues of roots and leaves (0.1 g) were homogenized in a 10% (*w*/*v*) TCA solution, and the samples were then centrifuged for 20 min at 12,000× *g* at 4 °C. The AsA content was measured spectrophotometrically at 265 nm. Three replicates were analyzed per treatment.

### 4.6. Determination of Enzyme Activity

The PME activity was determined according to published methods [53]. To assay the activity of PME, frozen samples (0.2 g) were ground and sequentially homogenized in a potassium phosphate buffer (50 mM) containing 1% PVP-30 and 1 mM EDTA. The samples were then centrifuged for 20 min (10,000× *g*, 4 °C), and the supernatant was collected and used for PME activity measurement. The GST activity was assayed as described by Singha et al. [88]. Briefly, frozen tissues of roots and leaves (0.2 g) were homogenized in a phosphate buffer, and the samples were then centrifuged for 10 min at 5000 rpm. The GST activity was calculated by using the extinction coefficient of the product formed. To assay the activity of PCS, frozen samples (0.2 g) were ground and sequentially homogenized in a buffer containing 100 μM CdSO_4_, 20% (*w*/*v*) glycerol, 100 mg mL^−1^ PVP, 10 mM β-mercaptoethanol, and 20 mM HEPES-NaOH (pH 7.5). The samples were then centrifuged for 10 min (13,000× *g*, 4 °C). The PCS activity was analyzed according to Wojas et al. [89]. Three replicates were analyzed per treatment.

To measure the activities of antioxidant enzymes, frozen tissues (0.5 g) were homogenized in a buffer containing 25 mM HEPES (pH 7.8), 2 mM ascorbate, 0.2 mM ethylenediaminetetraacetic acid (EDTA), and 2% (*w*/*v*) PVPP. Subsequently, the samples were centrifuged for 30 min at 12,000× *g* at 4 °C. Following centrifugation, the supernatants were collected and used for enzyme activity measurements. The SOD extraction buffer did not contain ascorbate. The SOD activity was determined by monitoring the inhibition of nitroblue tetrazolium (NBT) reduction at 560 nm, according to the method described by Goodarzi et al. [59]. The CAT activity was analyzed by determining the decomposition of H_2_O_2_ at 240 nm, according to an established method [90]. The POD activity was analyzed by the oxidation of pyrogallol, according to Qin et al. [91]. The activities of GR and APX were analyzed according to Jahan et al. [48]. The GR activity was measured with a kit (Solarbio Life Science, Beijing, China), according to the manufacturer’s protocol. To assay the activity of APX, frozen samples (0.2 g) were ground and sequentially homogenized in a potassium phosphate buffer (50 mM, pH = 7.0) containing 20 mM H_2_O_2_, 5 mM AsA, and 0.1 mM EDTA–Na_2_. The APX activity was estimated spectrophotometrically at 290 nm. Each treatment had three replicates.

### 4.7. Determination of Melatonin Content

The determination of endogenous melatonin content was performed by using HPLC, as described previously [92]. Samples (0.5 g) were ground in liquid nitrogen and extracted with methanol (5 mL). The samples were centrifuged for 30 min (10,000× *g*, 4 °C), and the combined supernatants were then dried using a nitrogen stream. Samples were dissolved in 200 μL of 0.1 M Na_2_HPO_4_: acetonitrile mixture (65:35), then subsequently filtered through a 0.22 μm filter. Five μL of the sample was injected into the C18 column (Shimadzu). The flow rate and the column temperature were 0.5 mL/min and 30 °C, with a detection wavelength of 220 nm. The endogenous melatonin content was calculated based on a standard curve. The melatonin standard was purchased from Sigma-Aldrich.

### 4.8. Statistical Analysis

Data analysis was performed using SPSS Statistics (version 22, SPSS Inc., Chicago, IL, USA). A one-way analysis of variance (ANOVA) followed by the least significant difference (LSD) test (*p* < 0.05) was used to compare the mean values between the different treatments. Graphs were drawn using Adobe Illustrator CS6, RStudio 3.6.1, and Sigmaplot 12.0.

## 5. Conclusions

Heavy metal toxicity severely affects crop growth and development, resulting in reduced crop productivity and deterioration in grain quality. As a pleiotropic molecule, melatonin exerts a vital role in regulating plant tolerance to heavy metal stress, such as Cr stress. Under Cr stress, melatonin enhanced the transcription levels of genes involved in pectin and hemicellulose biosynthesis (*UGDH*, *GAE*, *GAUT*, *CSL*, and *XYL*), resulting in increased cell wall polysaccharide contents. Meanwhile, melatonin increased the expression of genes involved in GSH and PCs biosynthesis (*SAT*, *Cysk*, *ATPS*, *APR*, *Sir* and *PCS*), leading to enhanced contents of GSH and PCs. The increased contents of cell wall polysaccharides, GSH, and PCs contribute to the retention of Cr in the cell wall and vacuole. Moreover, melatonin treatment alleviated Cr-induced oxidative stress by enhancing the activity of enzymatic and non-enzymatic antioxidants. Collectively, these findings indicate that melatonin alleviates Cr toxicity in maize by promoting Cr sequestration, re-establishing redox homeostasis, and inhibiting Cr transport from the root to the shoot. Altogether, this study provides insights into the mechanisms underlying melatonin-mediated Cr stress response in maize, with potential implications for crop production in contaminated soils.

## Data Availability

The data presented in this study were stored in the National Center for Biotechnology Information (PRJNA913565).

## Supplementary Materials

The following supporting information can be downloaded at: https://www.mdpi.com/article/10.3390/ijms24043816/s1.
