# Supplementary material for: Melatonin Alleviates Chromium Toxicity in Maize by Modulation of Cell Wall Polysaccharides Biosynthesis, Glutathione Metabolism, and Antioxidant Capacity
Source: Int J Mol Sci. 2023 Feb 14;24(4):3816. doi: 10.3390/ijms24043816 (PMC9966513; doi:10.3390/ijms24043816)
Supplement: Supplementary file 1 [file ijms-24-03816-s001.zip › Supplementary Materials.pdf]

Table S1. Melatonin and Cr induced changes in genes transcripts involved in heavy metal transport

Transcript_ID	Annotation	R-Cr VS R-C	R-Cr+M VS R-Cr
Zm00001d027884	*heavy metal ATPase2*	1.56	-1.23
GRMZM2G029951	*heavy metal ATPase4*	1.78	-1.09
Zm00001d015829	*heavy metal ATPase4*	2.31	-1.84

Table S3. Gene specific primers used in this study

Primer name	Transcript_ID or GenBank accession no.	Annotation	Forward	Reverse
ZmUbi-2-RT	NM_001329666.1	Polyubiquitin	TGGTTGTGGCTTCGTTGGTT	GCTGCAGAAGAGTTTTGGGTACA
ZmGAUT-RT	*Zm00001d009808*	*Galacturonsyl-transferase*	AGTTACTGCCTCTGGTCT	CACTCTGCCTTGTATGGA
ZmPME-RT	*Zm00001d005465*	*Pectin methylesterase*	GCAAGGACCTGCCCAAGAAC	CGGATGCCTCGGTGATGAG
ZmSAT-RT	*Zm00001d038737*	*Serine acetyltransferase*	AGTCGGAGGACGACGAGACC	GAGGAGGCAGTGGGAGAAGC
ZmGST-RT	*Zm00001d010870*	*Glutathione S-transferase*	GACCTCACGCTCTTTGAATCCC	GCGAAGCACTCCACCACGAT
ZmSOD-RT	*Zm00001d029170*	*Superoxide dismutase*	GGTGAAGGCTGTTGCTGT	CTCTGCCAATGATTGAGTTT
ZmAPX-RT	*Zm00001d023582*	*Ascorbate peroxidase*	CAAGAACATTACCGAGTGG	CTGGCAAGCTGAAACAGA
ZmNR-RT	*Zm00001d031494*	*Ascorbate peroxidase*	TTTTCTGATTCCTCGTGTT	TCTACTCTTCCTAGCCTTACTG
ZmTDC-RT	*Zm00001d024664*	*Tryptophan decarboxylasee*	TTCTTCCCGTCCACCAACAG	GGAGACAAGCGTGACGAGCAT

Maize experiment

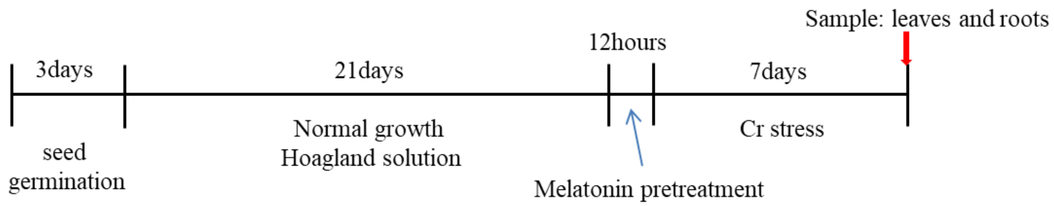

Arabidopsis thaliana experiment

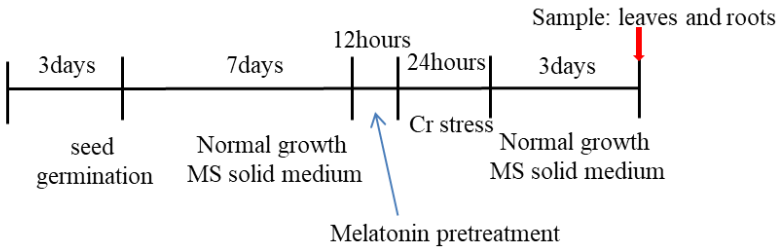

Figure. S1 The scheme of the experiment
